# *Plasmodium falciparum var* Gene Expression Homogeneity as a Marker of the Host-Parasite Relationship under Different Levels of Naturally Acquired Immunity to Malaria

**DOI:** 10.1371/journal.pone.0070467

**Published:** 2013-07-29

**Authors:** George M. Warimwe, Mario Recker, Esther W. Kiragu, Caroline O. Buckee, Juliana Wambua, Jennifer N. Musyoki, Kevin Marsh, Peter C. Bull

**Affiliations:** 1 Pathogen, Vector and Human Biology Department, Kenya Medical Research Institute-Wellcome Trust Research Programme, Kilifi, Kenya; 2 The Jenner Institute, University of Oxford, Oxford, United Kingdom; 3 Department of Zoology, University of Oxford, Oxford, United Kingdom; 4 Department of Epidemiology, Harvard School of Public Health, Boston, Massachusetts, United States of America; 5 Nuffield Department of Clinical Medicine, University of Oxford, Oxford, United Kingdom; University of Copenhagen, Denmark

## Abstract

Acquired immunity to *Plasmodium falciparum* infection causes a change from frequent, sometimes life-threatening, malaria in young children to asymptomatic, chronic infections in older children and adults. Little is known about how this transition occurs but antibodies to the extremely diverse PfEMP1 parasite antigens are thought to play a role. PfEMP1 is encoded by a family of 60 *var* genes that undergo clonal antigenic variation, potentially creating an antigenically heterogeneous infecting population of parasites within the host. Previous theoretical work suggests that antibodies to PfEMP1 may play a role in “orchestrating” their expression within infections leading to sequential, homogeneous expression of *var* genes, and prolonged infection chronicity. Here, using a cloning and sequencing approach we compare the *var* expression homogeneity (VEH) between isolates from children with asymptomatic and clinical infections. We show that asymptomatic infections have higher VEH than clinical infections and a broader host antibody response. We discuss this in relation to the potential role of host antibodies in promoting chronicity of infection and parasite survival through the low transmission season.

## Introduction

*P. falciparum* is a major cause of morbidity and mortality in sub-Saharan Africa [1], mainly in children. This age distribution of the disease burden can be attributed to the acquisition of immunity to malaria following prolonged exposure to infections. Substantial resistance to severe, life-threatening illness develops after relatively few exposures to the parasite [2]. However, immunity to mild malaria takes many years to develop and though older children and adults rarely suffer clinical attacks, they remain susceptible to chronic asymptomatic infections [3], [4]. This non-sterilizing resistance to disease suggests that naturally acquired immunity reflects an altered host-parasite interaction. Here, we examined the expression patterns of the large family of parasite molecules, PfEMP1 (*P. falciparum* erythrocyte membrane protein 1), which are thought to play a key role as targets of naturally acquired immunity.

PfEMP1 are encoded by about 60 *var* genes per parasite genome and expressed on the infected erythrocyte (IE) surface where they mediate cytoadhesion of IE to host receptors such as ICAM1 and CD36 [5], [6]. Following an episode of malaria individuals develop antibodies that are highly specific to the IE surface antigens expressed by each individual infection [7]–[11], but which have the potential to mediate variant-specific protection against future disease [5], [12]–[14]. In contrast, asymptomatic infections sampled during the low malaria transmission season are associated with an antibody response to a broad range of IE surface antigens when compared to individuals sampled at the same time that do not carry patent infections [15]–[18]. Notably, this association is evident even after allowing for differences in exposure, suggesting that these IE antibodies may be short-lived, broadly reactive responses to the current infection [15], [19]. Carriage of IE surface antibodies among children with ongoing asymptomatic infections is associated with protection from future clinical malaria episodes [15]–[18]. Despite this apparent importance of antibodies, the details of transition between clinical disease and asymptomatic infection are still unclear.

Here, we used a cloning and sequencing approach to compare the homogeneity of expression of *var* genes among parasites from 250 children with clinical and asymptomatic infections.

## Results

**Figure 1:**
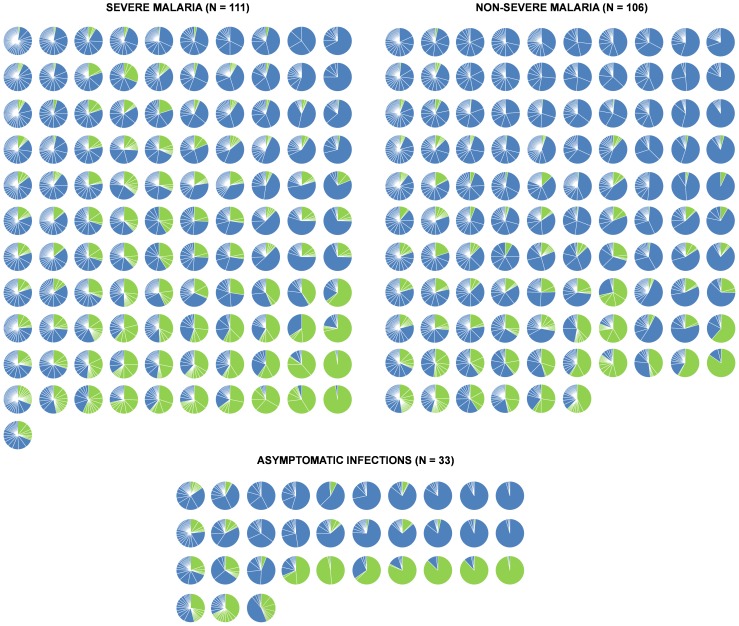
VEH and group A-like *var* expression levels among isolates from clinical and asymptomatic infections Presented are pie charts summarizing the *var* expression profiles of all isolates from children with clinical malaria (grouped into severe and non-severe malaria) and those with asymptomatic infection. Each slice of a pie chart (whether blue or green) represents a unique *var* sequence with green representing group-A like *var* sequences. The size of the slice represents the percentage contribution of that sequence to the entire *var* expression profile of the isolate. The pie charts are ordered from left to right by increasing VEH and top to bottom by increasing group-A like *var* expression levels.

Published *var* gene expression profiles from 217 isolates from children with clinical malaria and 33 with asymptomatic infection were used for this study [20], [21]. These were generated by sequencing, classifying and counting samples of short sequence tags amplified from the *var* DBLα domain region from parasite cDNA libraries prepared from each isolate. Since most PfEMP1 contain a single DBLα domain, this region is suitable for *var* gene expression profiling. Figure 1 summarizes the relative proportion of different sequences sampled from each isolate. Visual inspection of this data suggested that asymptomatic infections have more homogeneous expression profiles. To analyse this further we defined a measure of the homogeneity of the *var* expression profile based on Simpson’s diversity index ([22], see Materials and Methods).

### *Var* Expression Homogeneity (VEH) is Associated with Asymptomatic Infections

**Figure 2:**
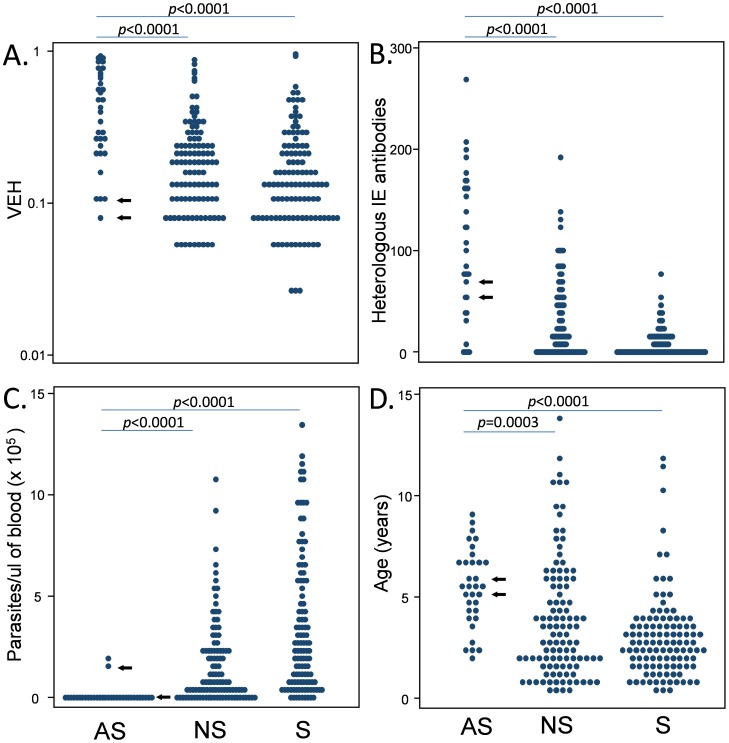
Comparisons of VEH, IE surface antibody breadth, parasitaemia and host age. The Mann-Whitney U test is used to compare asymptomatic and clinical infections in relation to VEH (A); heterologous IE surface antibody breadth measured as the median of the mean fluorescence intensities measured against eight different parasite isolates as described previously [21] (B); parasitaemia (parasites×10^5^/ µl blood, C); host age (D). Blue arrows are used to indicate two individuals (46C and 46D) who became symptomatic with within the two days of sampling.

Figure 2A compares the VEH in parasites from children with asymptomatic infections with those from children with clinical infections, both severe and non-severe. Significant differences were observed between asymptomatic and symptomatic infection (Fig. 2A). Other characteristics of these children are also shown in this figure. Figure 2B shows a measure of the breadth of their IE surface antibody response measured against 8 isolates by immunofluorescence [21] (see below). Children with asymptomatic infection had strikingly higher breadth of IE surface antibodies using this assay (Fig. 2B). Asymptomatic children also had significantly lower parasitaemia (Fig. 2C) and were older than symptomatic children (Fig. 2D).

Since RNA was extracted from 100 µl of packed RBC from each patient, the difference in VEH between asymptomatic and clinical infections was unlikely to be simply due to sampling fewer parasites from the asymptomatic children. However, to exclude the possibility of a relationship between number of parasites sampled and the observed VEH, serial parasite dilutions were performed on a single lab-adapted isolate (3D7) into the range of parasitaemia seen in asymptomatic infections. For each dilution, we synthesized cDNA and generated *var* sequence tags. We then assessed the relationship between the parasitaemia and VEH. If high VEH was an artifact caused by sampling low numbers of parasites from an isolate we would have expected VEH to be highest where the parasitaemia was most diluted. However, we found no evidence for this (Spearman’s rank correlation rho = −0.05, *p = *0.9, Fig. S1A). To show that results were consistent, sequencing of eight isolates from children with asymptomatic infections was repeated using freshly synthesized cDNA. Duplicate VEH scores were highly correlated (Spearman’s rank correlation rho = 0.88, *p = *0.004, Fig. S1B). Four of the original *var* profiles from these isolates, 31E, 45B, 45E and 46B, had clearly dominant sequences. The dominant sequences were identical when the cDNA synthesis and sequencing were repeated.

We considered the possible influence of the number of dominant parasite genotypes present in the infections on *var* expression homogeneity. The number of dominant genotypes was estimated by PCR amplification of MSP1 and GLURP genes using non-nested primers to MSP1 and GLURP, followed by visualization by gel electrophoresis. The maximum number of bands observed between the two primer sets was recorded. A non-nested approach was taken because, as with the *var* gene expression profiles, we were interested in the extent to which a small number of genotypes dominated the infection at the time of sampling rather than an estimate of the total number of genotypes causing the infection. Though the number of dominant genotypes showed an expected weak correlation with VEH (Spearman’s rank correlation rho = −0.14, *p = *0.03), this was unlikely to be important since the number of genotypes did not differ between asymptomatic and clinical infections (Mann-Whitney U test, *p = *0.95, mean number of dominant genotypes for asymptomatic: 1.6 [95% CI,1.3–1.8]; non-severe: 1.6 [95% CI,1.5–1.8]; severe: 1.5 [95% CI,1.4–1.7]). Furthermore, the difference in VEH between asymptomatic and clinical infections was evident even when considering infections where a single genotype was observed (Mann-Whitney U test, *p<*0.0001, comparing 18 asymptomatic and 116 clinical infections).

In further support of a difference between asymptomatic and clinical infections, two of the asymptomatic individuals (46C and 46D) attended hospital with clinical malaria within two days of sampling (indicated with arrows in Fig. 2). Parasites from these two individuals exhibited low VEH suggesting that diversification of *var* expression may be a characteristic of within-host parasite populations that are in the process of expanding.

### The Role of Host Antibodies in Shaping the Infecting Parasite Population

**Figure 3:**
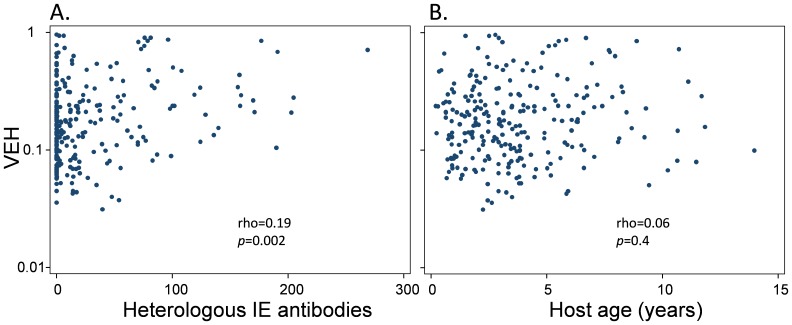
The relationship between VEH and host IE surface antibodies Scatter plots are shown of the relationship between VEH and the breadth of the heterologous IE surface antibody response (A, see legend to figure 2); (B) shows the relationship between VEH and host age. Spearman’s rank correlation coefficient rho was used to test for correlation.

We and others have previously proposed that antibodies impose a selection pressure on PfEMP1 molecules expressed by the infecting parasite population such that only transcripts encoding PfEMP1 variants to which the host lacks antibodies are expressed [12], [13]. Following from this we would predict that, in addition to the previously described shift in expression away from the group of *var* genes termed “group A-like” [21], [23], a broad repertoire of IE surface antibodies would be associated with more homogeneous *var* expression profiles in the infecting parasite population. Consistent with this prediction, the breadth of IE surface antibodies, measured for each child by immunofluorescence against 8 isolates [21], showed a positive correlation with VEH (Fig. 3A). Despite an association between host age and IE surface antibody breadth (Spearman’s rank correlation rho = 0.44, *p<*0.0001), host age showed no association with VEH (Fig. 3B).

**Figure 4:**
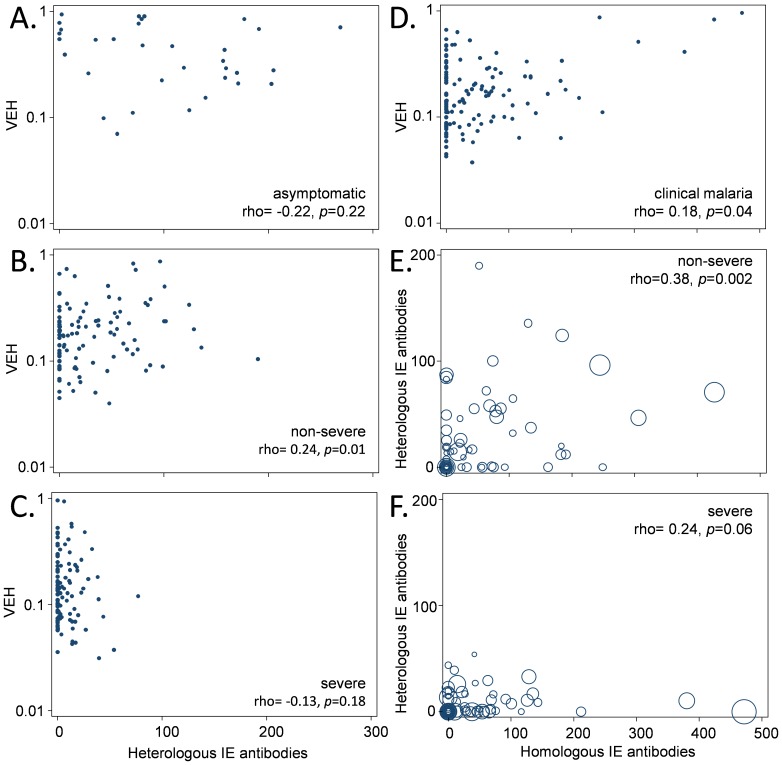
The relationship between VEH and host IE surface antibodies to heterologous and homologous isolates. A-C show scatter plots of the relationship between VEH and breadth of the heterologous IE surface antibody response as in Figure 3A, but split into asymptomatic (A), non-severe (B) and severe malaria (C). (D) Shows the relationship between VEH and the homologous IE surface antibody response to each child’s own parasites among children with malaria (measured as mean fluorescence intensity). Among children shown in (D), (E-F) compares the heterologous and homologous responses among those with non-severe (E) and severe (F) malaria, respectively. The size of the marker in (E-F) is proportional to VEH. Spearman’s rho was used to test for correlation.

The association with antibodies may suggest that antibodies play a role in determining the diversity of PfEMP1 expressed at any one time during an infection. However, the antibody breadth measure that we have used clearly does not explain all the variation in VEH. This is made clear when asymptomatic and symptomatic infections are viewed separately (Fig. 4A–C). Within the asymptomatic infection and severe malaria groups there was no evidence for a relationship between antibody breadth and VEH (Fig. 4A,C). However a significant positive relationship between antibody breadth and VEH was observed among parasites from non-severe malaria cases (Fig. 4B). Though this is consistent with a role for antibodies in shaping VEH, other host selection pressures or features of the parasites or their expressed PfEMP1 may also be influencing VEH within these groups of children. Since rosetting is associated with high parasitaemia [21], [24], we considered whether this phenotype may enable parasites to out compete other variants leading to higher VEH. However, there was no correlation between the percentage of parasites that formed rosettes and VEH (Spearman’s rank correlation rho = 0.06, *p = *0.52, n = 133).

Since our measure of IE surface antibody breadth is against eight parasite isolates, it only provides an estimate of the repertoire of variant specific antibodies carried by each individual. It provides no direct information on the ability of each individual to recognize and control the variants expressed within their own infection. Sufficient parasite material was available from 131 symptomatic children to explore the relationship between VEH and the “homologous” antibodies induced to the infecting parasite population. We tested each of the 131 parasite isolates against each child’s homologous acute plasma sampled at the time of disease (see Table S2). A weak positive association was observed between the homologous IE surface antibody response and VEH (Fig. 4D, Spearman’s rank correlation rho = 0.18, *p = *0.04). This experiment still has clear limitations because we do not have information about recognition of other variants previously expressed by the infecting parasite population other than those expressed at the time of sampling. However, the results are consistent with the idea that antibodies are involved in limiting the diversity of PfEMP1 expressed by the parasite population. There was no evidence that either homologous IE antibodies or IE antibody breadth were associated with the number of dominant genotypes present (Spearman’s rank correlation rho = −0.0003, *p* = 1.00 and rho = 0.02, *p* = 0.73 respectively).

An alternative interpretation is that some variants are efficient at evading opsonization and maintaining cytoadherence even in the presence of a strong antibody response. Indeed recent theoretical work has explained the evolution of a subset of long *var* genes, such as those in group A, by predicting that they encode PfEMP1 that are able to mediate cytoadherence with host molecules and maintain sequestration and survival of parasites even in the presence of host antibodies by engaging alternative cytoadherence domains that are not recognized by the host [25]. It was notable that among the 131 children with acute plasma available, the child with the highest homologous antibody response (27B) also had the highest VEH, which was mainly attributable to a single group A-like *var* gene that constituted 98% of the *var* genes expressed by the parasite isolate (Figure S2). Whilst no conclusive interpretations can be made based on this data the hypothesis by Buckee and Recker [25] warrants further study.

## Discussion

Chronic asymptomatic infections are an essential part of the parasite life cycle because they enhance parasite survival during the dry seasons when the mosquito vector is absent and transmission is low. However, the way in which parasites are able to survive within the host for such long periods without exhausting their variant antigen repertoire or causing clinical symptoms is poorly understood. A possible explanation is that the host-parasite interaction in chronic, asymptomatic infections is fundamentally different from that in clinical malaria. This is supported by the fact that asymptomatic infections sampled at a single time point at the end of a period of low malaria transmission are associated with lower parasite densities and broader IE surface antibody repertoires when compared to clinical infections. Here, we further show that isolates from children with asymptomatic infection have more focused expression of *var* genes (higher VEH) than those from children with clinical malaria. One weakness of this study is that we sampled relatively few asymptomatic infections and only at a single time point. Further studies are clearly needed to confirm that our observations are generally true over the duration of chronic infections.

**Figure 5:**
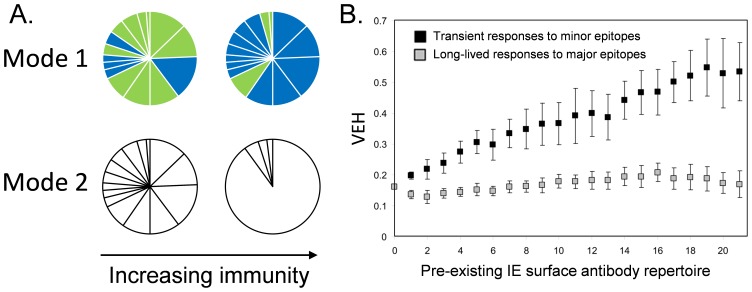
Modeling the relationship between VEH and host IE surface antibodies. In (A) we use pie charts to represent two modes in which acquired immunity may modify the parasite population in a single host. In “mode 1” IE surface antibodies alter the relative abundance of parasites expressing distinct PfEMP1 groups (pie chart slices, colours blue and green). In “mode 2” the acquired IE surface antibody repertoire is able to control a wide range of PfEMP1 variants such that only a small number of variants dominate the PfEMP1 expression profile of the infecting parasite population at any one time. Presented in (B) is an output from the mathematical model by Recker *et al.* This model was originally used to emphasize how a combination of long-lived antibodies to unique major epitopes and transient antibodies to shared minor epitopes can limit the number of synchronously expressed variants and prolong an infection [19]. Here, we replaced the duration of infection with a measure of the mean VEH of the parasite population following runs of the model with different initial levels of antibodies to major and minor epitopes. The aim was to simulate infections at different stages of their development. The graph shows the relationship between the initial number of each type of antibody [19] and mean VEH. We speculate that the ability of the host to induce transient partially cross-reactive antibodies in the model is equivalent to the observed breadth of IE surface antibody responses described in our dataset. Error bars represent the standard deviations.

We and others have previously shown that expression levels of group A-like *var* genes are negatively associated with both host age [20], [26] and IE surface antibodies [20] [21]. We suggest that this may be one of two ways in which naturally acquired immunity is acquired (Fig. 5A). On the one hand, IE surface antibodies impose a negative selection pressure on specific broad groups of PfEMP1(Mode 1, Fig. 5A). The results presented here add support to a second mode of immunity, in which the host is able to limit the diversity of the PfEMP1 sequence types expressed by the infecting parasite population (Mode 2, Fig. 5A). The results are consistent with previous studies showing that antibodies impose a variant specific selection pressure on variant surface antigens expressed during disease [12] [13].

Much theoretical work has focused on the maintenance of chronic asymptomatic infections through sequential antigenic variation [27]. Two major problems arise when attempting to explain sequential switching in *P. falciparum*. First, the repertoire of PfEMP1 is relatively small. Second, the switch rate between variants is high; 2% to 18% switch rates have been reported [28], [29]. It is therefore puzzling how chronic asymptomatic infections are sustained without rapidly exhausting the genomic PfEMP1 repertoire. Two recent theoretical models have suggested the importance of non-variant specific antibodies in controlling parasitaemia and limiting the rate of repertoire exhaustion. One incorporates antibodies to non-PfEMP1 molecules but will still only work if parasites have low switch rates [30]. Another is compatible with higher switch rates and involves short-lived antibody responses with a limited level of cross reactivity to minor PfEMP1 epitopes coupled with long lived, variant specific responses to major epitopes [19]. The effect of the partially cross-reactive antibodies in this latter model is to restrict the variants capable of dominating at any one time, equivalent to an increase in VEH described here. Successive waves of variants are those few that are recognized neither by long-lived responses to major epitopes nor the current wave of cross-reactive responses to minor epitopes. The efficiency of the responses to minor epitopes was shown to correlate positively with the duration of the infection and negatively with peak parasitaemia [19]. It is possible that differences in the efficiency of responses to minor and major epitopes exist between individuals with clinical and asymptomatic infections. Alternatively, it may simply be necessary to build up a threshold number of responses to minor epitopes before a chronic infection can be maintained. Indeed, using the model previously developed in [19] we found that VEH is predicted to rise in relation to the number of responses accumulated to minor epitopes (Figure 5B). If responses to minor epitopes lead to an increase in the breadth of measured antibodies to several isolates, the observed positive correlation within the theoretical model is consistent with the data presented here, although there is clearly considerable variation in VEH that cannot be explained solely by this model (Figure 4A–C).

Parallel studies with laboratory isolates have raised the possibility that parasites have partitioned their variant antigens into those with low and high switch rates [31]. Modeling studies of such parasites show that infecting parasite populations expressing variants with different switching characteristics would potentially lead to alternative paths within the network of possible switches. Immune selection could lead to diversion from a path in which many variants are expressed to one in which only small numbers of variants are expressed. As suggested by Turner [32] in relation to *Trypanosoma brucei* infections, rapid switching early in infection would enhance competition between different genotypes, whilst slower switch rates later in infection would extend infection length. Such a model is compatible with studies of parasite infected volunteers following mosquito-inoculation in which many parasite variants dominate early in an infection, suggesting that the first wave of parasites explore the available immunological space of the host rather than committing to specific variants to which the host may already have immunity to [33]. A recent study in *Plasmodium chabaudi* also shows that parasites recently transmitted to mice by mosquitoes express a much broader repertoire of *cir* variant antigen genes than parasites that have been serially passaged in mice [34].

We have presented preliminary evidence supporting a model that involves host immune selection, although further studies are needed to fully establish the relative roles of host selection and parasite control of *var* switching in explaining the variation in VEH between isolates. Indeed, a study of parasites from The Gambia [35] suggests an association between elevated PfSir2 expression and severe malaria, which may involve a dysregulation of the epigenetic control of *var* expression. This raises the possibility that parasites may modulate their switching behavior in response to the host or as part of a developmental programme.

## Materials and Methods

### Ethics Statement

Ethical approval for this study was granted by the Kenya Medical Research Institute (KEMRI) Ethical Review Committee, and written informed consent was obtained from the parents/guardians of all study participants.

### Study Site

The study was carried out at Kilifi District Hospital, situated at the coast of Kenya. Ethical approval for this study was granted by the Kenya Medical Research Institute Ethical Review Committee and informed consent obtained from the parents/guardians of all study participants.

### Parasite Sampling

Details of the isolates from children with clinical malaria and asymptomatic infections were published previously [20], [21]. Of the clinical cases severe malaria was defined as hospital admission with impaired consciousness (Blantyre coma score <4 in patients under 8 months old; <5 in patients aged ≥8 months), severe malarial anemia (hemoglobin <5 g/dl), or respiratory distress (deep “Kussmaul” pattern of breathing). These were sampled between August 2003 and September 2007. Peak malaria transmission in Kilifi follows two rainy seasons in May to July and November to December. Isolates from asymptomatic infections of any parasite density were sampled during a cross-sectional survey just before the start of the rainy season in May 2007 and are therefore most likely to be from chronic infections that had been carried since the last transmission season. These children have since been monitored for episodes of malaria as part of an ongoing study.

### *Var* Expression Profiling

For each child, expressed *var* sequences were generated from ring-stage parasites as described previously [23] and are accessible in the EMBL nucleotide sequence database (EMBL accession numbers FN588437–FN592661; [20] for the symptomatic cases and HE654181–HE654544 for the asymptomatic cases). Sequence assembly, classification and counting were done using two previously described analysis pipelines [20], [36]. These analysis pipelines were modified to calculate each isolate’s Simpson’s Index of diversity as described below [22]. Expression frequencies of each sequence tag are given in [21] for clinical cases and Table S1 for asymptomatic cases.

### Parasite Genotyping

Parasite DNA was extracted either from whole EDTA treated blood using a QIAamp DNA Blood Mini Kit (Qiagen) or obtained as residual DNA in extracted RNA preparations using Trizol that were performed on white cell depleted, heparinized blood as described in ref [23]. MSP1 and GLURP genes were amplified as described previously [37], [38]. Bands were visualized using electrophoresis on 2% agarose gels.

### *Var* Expression Homogeneity (VEH)

We defined VEH as the extent to which a small number of *var* gene sequences dominate an isolate’s expression profile. In a previous preliminary study we attempted to capture this using the percentage of clones from each isolate that were among the most dominant two sequences [23]. Here, to fully capture the homogeneity of the *var* genes expressed by each isolate we calculated the Simpson’s Index of diversity (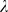) [22], defined here as the sum of the squares of the frequencies of each sequence type represented in the var expression profile, (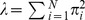, where 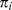 represents the proportion of a distinct *var* gene sequence *i* among all N clones sampled from an isolate). The value of 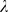 thus ranges between 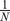, representing the least homogeneous *var* expression profile (lowest VEH), and 1, representing the most homogeneous *var* expression profile (highest VEH). This is the basic form of the original Simpson’s diversity index, which does not take into account the number of sequences sampled. We used this because the number of sequences sampled per isolate was within a range that was unlikely to influence the final calculation, which increases when the number of sequences sampled per isolate is very low. The mean number of sequences sampled per isolate was 68.0 (95% CI, 66.0–69.0). The number of sequences sampled in each patient group was very similar, and if anything slightly higher in the asymptomatic cases: asymptomatic, mean, 73.0 (95% CI, 69.3–76.6); non-severe, 67.6 (95% CI, 65.0–70.2); severe, 66.2 (95% CI, 63.5–68.9). See table S1 in ref. [21].

### Assessment of Host IE Surface Antibodies

For each child’s plasma, flow cytometry was used to determine IgG antibody levels against the infected erythrocyte surface of 8 clinical isolates. These heterologous IE antibody data are published elsewhere [20], [21]. For each child, the plasma used was collected at the same time as the corresponding isolate used for sequence analysis. To estimate each child’s IE surface antibody breadth the median of each child’s responses to the 8 isolates (acquired as mean fluorescence intensity, MFI) was used. However, for three children with antibody data available for only five of the 8 isolates the antibody breadth score was based on the five isolates. Homologous IE antibodies were measured for a subset of children’s plasma using the method described previously [20], [21] except plasma were tested against cultured parasites from the same child. After sampling from the patient, parasites were cultured until they matured to mid to late trophozoite stage and stored frozen as described in [21].

### Statistical Analysis

The statistical software Stata™ version 11 was used for all the analysis and *p* value <0.05 considered significant for all tests. Spearman’s rank correlation coefficient, rho was used to assess the relationship between VEH and serial parasitaemia dilutions of a single isolate (Fig. S1), host antibodies (Figs. 3–4) and host age (Fig. 3B). The Mann-Whitney U test was used to compare clinical and asymptomatic infections with respect to: IE surface antibody breadth (Fig. 2B), host age (Fig. 2D), parasitaemia (Fig. 2C), VEH (Fig. 2A) and multiplicity of infection (see text).

#### Mathematical model

We used the mathematical model described by Recker *et al.* [19] to explore how orchestration of antigenic variation might lead to an increase in the homogeneity of *var* expression (VEH) by parasites sampled at a single time point. In this model, which is described as a set of differential equations in [19], transient, partially cross reactive antibodies are shown to be capable of limiting the number of variants drawn at any one time from a set of hypothetical variants. We investigated the simulated relationship between parasite VEH and the host’s pre-existing antibody repertoire generated by this model by randomly assigning different numbers of cross-reactive responses to minor epitopes or variant specific immune responses to major epitopes at non-zero levels at the start of the infection (Fig. 5B). The model uses 42 variant antigen molecules, each with a single unique epitope and two minor epitopes 1 and 2. Minor epitope 1 had 6 variant forms and minor epitope 2 had 7 variant forms. As the variant distribution during the initial peak is strongly dependent on the initial conditions within this framework, we calculated an average VEH over a fixed period of time instead (*t = *200 arbitrary units); this also takes into account the uncertainty about the onset of infection in the data and is therefore better suited for qualitative comparisons. VEH within this framework was then calculated as 
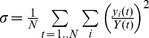
, where *y_i_*(*t*) is the frequency of variant *i* at time *t* and *Y*(*t*) is the sum of all variants, i.e. 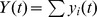. The model was run 200 times to obtain mean VEH scores and standard deviations.

## Supporting Information

Figure S1 **Reproducibility of the VEH assay.** A) Parasitaemia dilutions in a single isolate 3D7 are shown in relation to VEH. The parasitaemia dilutions were done using freshly prepared blood group O cells before synthesizing cDNA from 100 µl packed IE and generating *var* sequence tags. The interquartile range of parasitaemias observed in the asymptomatic patients was 0.1%–0.8% IE. B) repeat measures of VEH using freshly prepared cDNA is compared with the original VEH estimate. Repeat measures were generally lower because they were calculated from raw sequence data from single reads for each bacterial colony picked, whereas original VEH measures were calculated from assembled sequence data which will tend to collapse sequencing errors into fewer consensus sequences. Spearman’s rank correlation coefficient and *p* values are indicated.
(TIF)

Figure S2 **Heterologous and homologous IE surface antibody response in relation to expression of group A-like var genes.** The heterologous and homologous responses are compared as in Figure 4 (C-D). The size of each marker is proportional to the expression levels of group A-like genes.
(TIF)

Table S1 (XLSX)

Table S2 (XLSX)
