# Supplementary figures and images for: Plasmodium falciparum var Gene Expression Homogeneity as a Marker of the Host-Parasite Relationship under Different Levels of Naturally Acquired Immunity to Malaria
Source: PLoS One. 2013 Jul 29;8(7):e70467. doi: 10.1371/journal.pone.0070467 (PMC3726600; doi:10.1371/journal.pone.0070467)

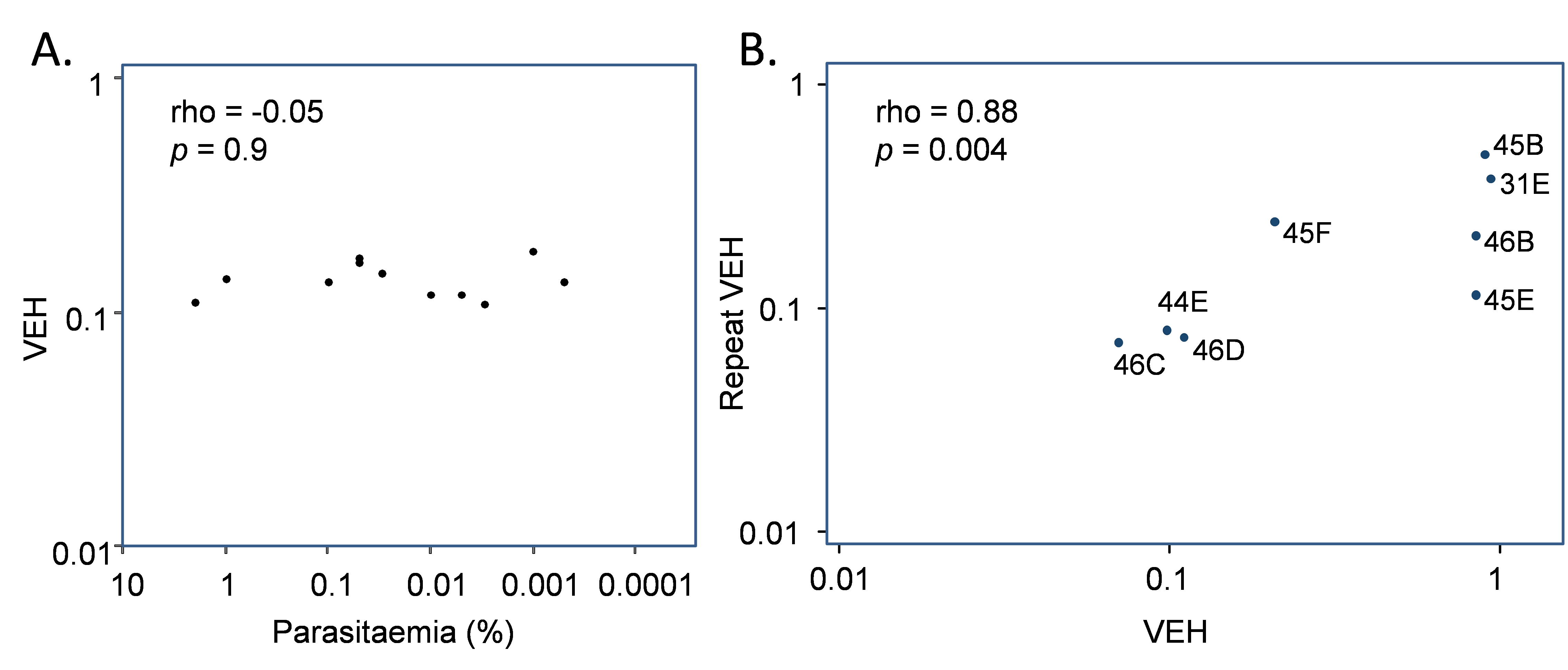

Supplement: Figure S1 — Reproducibility of the VEH assay. A) Parasitaemia dilutions in a single isolate 3D7 are shown in relation to VEH. The parasitaemia dilutions were done using freshly prepared blood group O cells before synthesizing cDNA from 100 µl packed IE and generating var sequence tags. The interquartile range of parasitaemias observed in the asymptomatic patients was 0.1%–0.8% IE. B) repeat measures of VEH using freshly prepared cDNA is compared with the original VEH estimate. Repeat measures were generally lower because they were calculated from raw sequence data from single reads for each bacterial colony picked, whereas original VEH measures were calculated from assembled sequence data which will tend to collapse sequencing errors into fewer consensus sequences. Spearman’s rank correlation coefficient and p values are indicated. (TIF) [file pone.0070467.s001.tif]

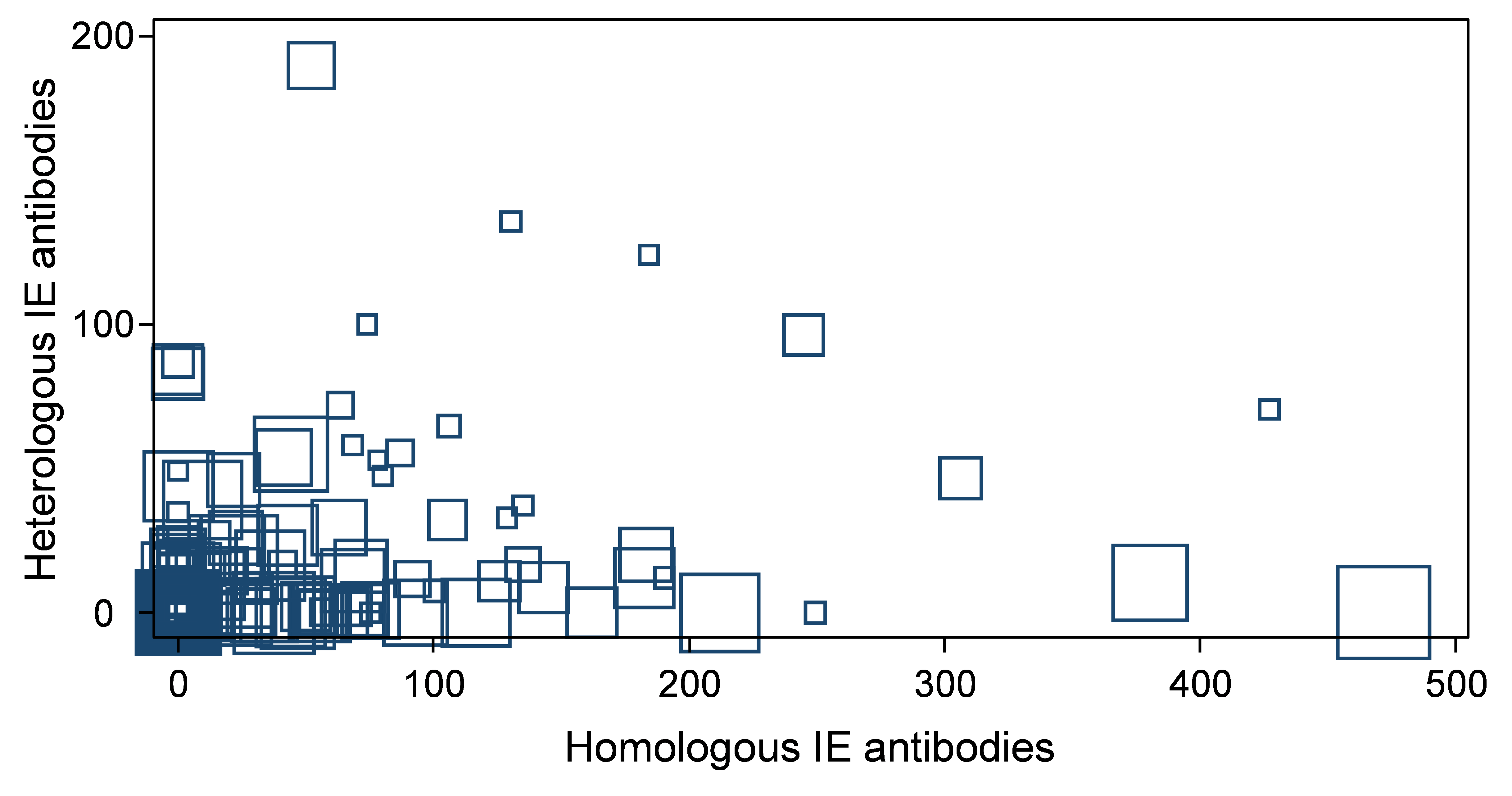

Supplement: Figure S2 — Heterologous and homologous IE surface antibody response in relation to expression of group A-like var genes. The heterologous and homologous responses are compared as in Figure 4 (C-D). The size of each marker is proportional to the expression levels of group A-like genes. (TIF) [file pone.0070467.s002.tif]
